# Metabolic Cancer-Macrophage Crosstalk in the Tumor Microenvironment

**DOI:** 10.3390/biology9110380

**Published:** 2020-11-07

**Authors:** Kyra E. de Goede, Amber J. M. Driessen, Jan Van den Bossche

**Affiliations:** Department of Molecular Cell Biology and Immunology, Amsterdam UMC, Vrije Universiteit Amsterdam, Cancer Center Amsterdam, 1081 HV Amsterdam, The Netherlands; k.degoede@amsterdamumc.nl (K.E.d.G.); amber.driessen@student.uva.nl (A.J.M.D.)

**Keywords:** tumor, macrophages, metabolism, oncometabolite, TAM, tumor-associated macrophage, cancer

## Abstract

**Simple Summary:**

Tumors often include many immune cells that are theoretically able to combat tumor growth. Yet, tumors can induce immune functions that support tumor growth via different routes. In this review, we discuss how cancer cell metabolism regulates the activity of macrophages within tumors and how this affects tumor progression. This is particularly relevant as metabolic pathways in both cancer and immune cells can serve as targets to improve cancer treatment.

**Abstract:**

Tumors consist of a wide variety of cells, including immune cells, that affect tumor progression. Macrophages are abundant innate immune cells in the tumor microenvironment (TME) and are crucial in regulating tumorigenicity. Specific metabolic conditions in the TME can alter the phenotype of tumor-associated macrophages (TAMs) in a direction that supports their pro-tumor functions. One of these conditions is the accumulation of metabolites, also known as oncometabolites. Interactions of oncometabolites with TAMs can promote a pro-tumorigenic phenotype, thereby sustaining cancer cell growth and decreasing the chance of eradication. This review focuses on the metabolic cancer-macrophage crosstalk in the TME. We discuss how cancer cell metabolism and oncometabolites affect macrophage phenotype and function, and conversely how macrophage metabolism can impact tumor progression. Lastly, we propose tumor-secreted exosome-mediated metabolic signaling as a potential factor in tumorigenesis. Insight in these processes may contribute to the development of novel cancer therapies.

## 1. Introduction

The tumor microenvironment (TME) harbors many components such as cancer cells, immune cells, fibroblasts, and endothelial cells. In 2011, an inflammatory TME was recognized as a hallmark of cancer [1]. Although immune cells are one of the most abundant cell types present in the majority of solid tumors, the TME is often immunosuppressive. The majority of the tumor is comprised of macrophages [2], which are theoretically capable of eliciting anti-tumor responses. However, an increased abundance of tumor-associated macrophages (TAMs) is generally associated with worse prognosis [3,4] as cancer cells have developed mechanisms to induce macrophages that promote tumor growth by regulating angiogenesis, promoting metastasis and suppressing immune function [5]. TAMs consist of heterogeneous subsets that are regulated by microenvironmental factors such as chemokines, cytokines, growth factors, but also metabolites [5,6].

Although TAMs are mostly considered pro-tumoral and occur predominantly in established tumors, TAMs can also demonstrate a pro-inflammatory phenotype, which particularly occurs in early stages [7]. However, secretion of pro-inflammatory cytokines and reactive oxygen species (ROS) by myeloid cells does not automatically lead to anti-tumor responses but can also contribute to tumorigenesis and metastasis, as cancer-associated inflammation is both a hallmark and a driver of cancer. Pro-tumorigenic functions of inflammation are exerted by increasing mutational load and cellular signaling resulting in DNA and epigenetic modifications [8]. Signaling by pro-inflammatory cytokines may also induce survival of transformed cells, activate other pro-tumorigenic stromal cells and induce epithelial-to-mesenchymal transition (EMT), a process required for cancer metastasis and correlated with worse prognosis [8]. Nevertheless, pro-inflammatory macrophages can phagocytose and present tumor-antigens, provide co-stimulatory signals and secrete cytokines activating T effector cells, and are associated with improved prognosis [9]. On the other hand, anti-inflammatory TAMs are mostly immunosuppressive, pro-metastatic through the remodeling of the extracellular matrix, and pro-angiogenic, thereby allowing cancer progression [8]. TAMs exert immunosuppressive functions by several mechanisms: expression of programmed death ligand 1 (PD-L1), thereby limiting T cell responses [10]; secretion of anti-inflammatory cytokines such as interleukin-10 (IL-10) and transforming growth factor β (TGFβ) [11]; recruitment of regulatory T cells (Tregs) via chemokine secretion [12]; and conversion of cluster of differentiation (CD) 4^+^ T cells towards Tregs [13]. For a more detailed description of the pro- and anti-tumorigenic roles of inflammation in cancer we refer to [8,14].

These pro- and anti-inflammatory TAM subsets not only differ in their phenotype regarding cytokine production and surface marker expression, but also in their metabolism. The specific metabolic state of macrophages is not only required to meet their altered energy and biosynthesis demands, but can also directly drive their function [15]. Pro-inflammatory macrophages rely on glycolysis, which in addition to ATP produces biosynthetic intermediates and fuels the pentose phosphate pathway for nucleotide synthesis and ROS and nitric oxide (NO) production [15]. Additionally, they have a truncated tricarboxylic acid (TCA) cycle preventing effective oxidative phosphorylation (OXPHOS) [16] and use the electron transport chain for reverse electron transport resulting in ROS rather than ATP production [17]. Pro-inflammatory macrophages also upregulate fatty acid synthesis to reorganize their cell membrane for inflammatory signaling [15]. On the contrary, anti-inflammatory macrophages show an intact TCA cycle and increased OXPHOS. Originally, fatty acid oxidation was thought to be crucial for this macrophage subtype; however, later glycolysis was recognized as an alternative pathway to fuel OXPHOS [15,18,19].

Tumors can exploit this connection between immune cell metabolism and function to suppress immunity and to promote tumor progression. Tumors exhibit altered metabolism compared to normal tissues, preferring glycolysis over OXPHOS even in the presence of oxygen, a phenomenon known as the Warburg effect [20,21]. This has the advantage of providing both biosynthetic building blocks needed for proliferation and signaling intermediates that can affect the phenotype of cells present in the TME [21]. This increased glucose consumption, in addition to the occurrence of hypoxic areas within the tumor, can lead to nutrient competition between cancer and immune cells. Moreover, altered metabolism and genetic mutations can result in aberrant production of metabolites such as lactate, succinate and 2-hydroxyglutarate (2HG), respectively [22,23,24]. These oncometabolites can interact with TAMs and affect their phenotype and ultimately tumor progression.

## 2. Metabolic Conditions Affect the TME

**Figure 1:**
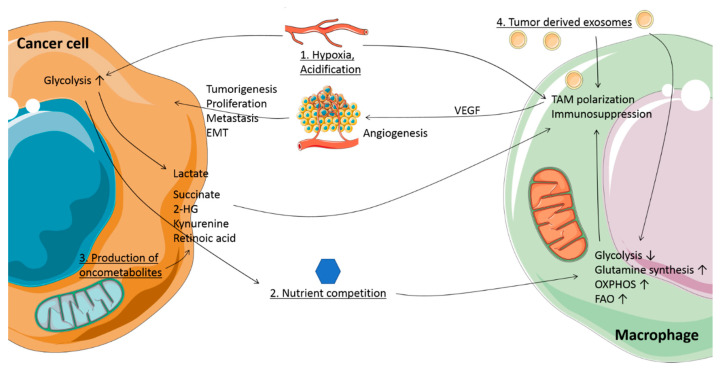
Metabolic conditions and crosstalk between cancer cells and macrophages in the tumor microenvironment (TME). 1. Hypoxia: the TME contains regions with reduced oxygen availability. Together with Warburg metabolism, this promotes lactate production in tumor cells by upregulation of glycolysis. Lactate is transported out of the cells and together with the concomitant acidification influences tumor-associated macrophage (TAM) phenotype. 2. Nutrient competition: presence of glucose and amino acids is limited due to the high metabolic rate of cancer cells, inducing metabolic changes in TAMs, and affecting their function. 3. Production of oncometabolites: mutations in tricarboxylic acid (TCA) cycle enzyme genes, as well as changes in metabolism, lead to accumulation of oncometabolites such as succinate, 2-hydroxyglutarate (2HG), lactate, kynurenine, and retinoic acid. When secreted, these oncometabolites support a pro-tumoral TAM phenotype and function. 4. Tumor-derived exosomes: tumors exhibit an increase in exosome release, which can carry microRNA, oncometabolites and metabolic enzymes, affecting macrophage function and metabolism. Together, these metabolic conditions and oncometabolites induce tumor-supporting TAMs, with functions such as promoting angiogenesis, metastasis, epithelial-to-mesenchymal transition (EMT), proliferation and immunosuppression.

Many solid tumors exhibit hypoxic areas due to rapid tumor growth and structural and functional abnormalities of the microvasculature [25]. Exposure to hypoxia stimulates cancer cell invasiveness and metastasis, increases resistance to therapy and decreases patient survival [26,27]. In order to withstand the cellular stress induced by hypoxia, cells respond by activation of the hypoxia-inducible factor 1α (HIF1α) pathway. HIF1α is an essential regulator of gene expression in response to oxygen-limiting conditions and upregulation of this pathway induces glycolytic genes [27]. This can lead to elevated lactate levels (Figure 1), which is transported out of the cell by HIF1α-induced upregulation of monocarboxylate transporter (MCT4) [28]. Interestingly, lactate produced by tumor cells in hypoxic areas can be imported via MCT1 and used as fuel for the TCA cycle by better oxygenated cancer cells [29]. The utilization of lactate by the latter saves glucose for glycolytic tumor cells in hypoxic regions and benefits the metabolism of both, also known as metabolic symbiosis [29]. High rates of lactate production furthermore lead to acidification of the TME, which can be sensed by macrophages and induce a pro-tumoral profile in TAMs (Figure 1) [30]. Additionally, hypoxic areas recruit angiogenic TAMs to improve vascularization and blood flow within the tumor. Consistently, high numbers of TAMs have been found in hypoxic areas of breast cancers in vivo [7,31], further contributing to the pro-tumorigenic microenvironment.

High glycolysis and limited nutrient supply due to disorganized vascularization result in competition for glucose between cancer and stromal cells. The TME has been shown to harbor low levels of glucose and glycolysis intermediates [32,33], affecting TAM metabolism (Figure 1). In vitro TAM models demonstrate upregulation of glycolysis [34,35,36], but also increased mitochondrial respiration, which may help survival in case glucose availability is insufficient [35]. However, another study found hypoxic TAMs in lung and breast cancer mouse models to upregulate regulated in development and DNA damage responses 1 (REDD1), an inhibitor of mammalian target of rapamycin (mTOR) and thereby decreasing glycolysis [37,38]. Reversing the latter by REDD1 deletion in these TAMs allows them to compete for glucose with endothelial cells, leading to improved blood vessel stability and decreased metastasis [37]. Furthermore, TAMs have been found to oxidize more lactic acid than other cells within a Lewis lung cancer (LLC) mouse model [22], indicating that a lack of glucose may force TAMs to switch to alternative fuel sources (Figure 1). Another scarce fuel source is glutamine [39]. TAMs in LLC mouse models and glioblastoma patients have been found to upregulate glutamine synthetase, which is induced in response to starvation and can elicit pro-tumorigenic TAM polarization [22,40,41]. A macrophage-specific knockout of glutamine synthetase reverses LLC-associated TAM polarization to an anti-tumoral phenotype, decreasing metastasis, normalizing vasculature and increasing CD8^+^ T cell infiltration [41]. Additionally, low levels of arginine and tryptophan have been detected in the TME of human brain, gynecological, kidney and lung cancers due to increased cancer cell expression of enzymes metabolizing these amino acids [42,43,44]. However, also high expression of arginase 1 (*Arg1*) in TAMs could contribute to low arginine concentrations [45], which is required for T cell survival and anti-tumor responses [45,46]. Similar to arginine shortage, tryptophan starvation has been implicated to induce a regulatory T cell phenotype [47].

Together, these metabolic conditions show the capability of tumor cells to adapt to a changing environment and thereby affect TAM metabolism and function. How TME characteristics translate into tumor cell-induced manipulation of TAMs towards a pro-tumorigenic phenotype, ultimately increasing chances of survival, will be discussed in a later chapter.

## 3. Oncometabolites Accumulate due to Mutations in TCA Cycle Enzyme Genes

Although cancer cells heavily rely on glycolysis, the tricarboxylic acid (TCA) cycle is still crucial [48,49]. Mutations in metabolic enzyme genes are a known risk factor for developing cancer [50]. Loss-of-function mutations in succinate dehydrogenase (SDH), present in human paragangliomas and pheochromocytomas, and fumarate dehydrogenase (FH), present in renal cell cancer patients, lead to accumulation of succinate and fumarate, respectively, while gain-of-function mutations in isocitrate dehydrogenase (IDH) produce the D-enantiomer of 2HG in human gliomas (D-2HG) [24,51]. These metabolites can support tumorigenesis [50]. Mutations of all four SDH subunit genes have been linked to cancer [52,53,54,55]. Succinate, fumarate and D-2HG aid tumor progression by epigenetic remodeling as they are structurally similar to α-ketoglutarate (αKG) and can therefore act as competitive inhibitors of αKG-dependent dioxygenases [56]. This mechanism results in aberrant HIF1α accumulation and DNA hypermethylation [50]. For instance, mutations in SDH subunit B (SDHB) induce the most epigenetic silencing in cancer cells, increasing cell migration and potentially explaining malignancy [57]. Furthermore, lactate, succinate, fumarate and 2HG have been shown to induce EMT via epigenetic regulation in murine tumor models and cancer patients [58,59,60,61]. Pro-tumorigenic oncometabolite signaling is reviewed in more detail here [50].

Both succinate and D-2HG can be secreted by cancer cells [23,62], exposing the TME to elevated levels of oncometabolites. Interestingly, the concentration of D-2HG correlates with tumor size and response to therapy in biliary tract cancer patients [62]. In summary, the TME experiences high levels of aberrantly accumulating oncometabolites due to mutations, such as succinate, fumarate or 2HG, or altered tumor metabolism, such as lactate and the tryptophan metabolite kynurenine. In the following section, we discuss their effects on TAMs.

## 4. Oncometabolites Regulate Macrophage Function and Heterogeneity

### 4.1. Cancer-Derived Lactate Induces TAMs that Promote Angiogenesis and Tumor Metastasis

**Figure 2:**
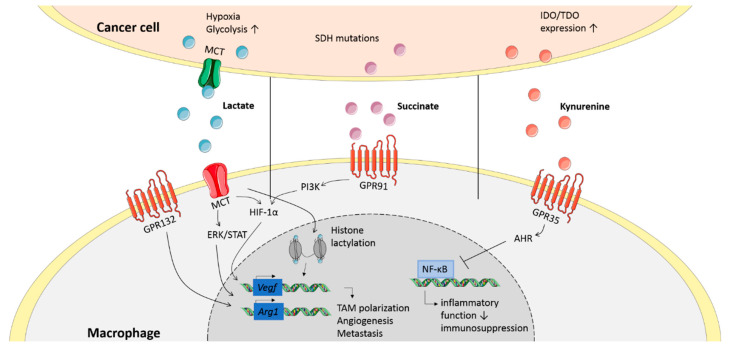
Signaling of oncometabolites in macrophages. Cancer cells secrete various oncometabolites, of which lactate, succinate and kynurenine are shown here. These oncometabolites signal through receptors or get internalized by transporters on the membrane of macrophages. This inhibits inflammatory functions and induces expression of genes related to an anti-inflammatory phenotype.

Lactate does not only affect tumor cells as described above but also macrophages. MCT4-mediated uptake of tumor-derived lactic acid increases vascular endothelial growth factor (*Vegf*) and *Arg1* expressions in murine TAMs, which substantially supports tumor growth (Figure 2) [22]. This was suggested to be HIF1α-mediated; however, recently, a different mechanism was proposed as lactate was found to directly alter gene transcription by histone lactylation, polarizing pro- to anti-inflammatory macrophages in vitro. Furthermore, histone lactylation is associated with higher *Arg1* expression in in vivo LLC and melanoma models, indicating a novel mechanism of TAM polarization [63].

In addition to the effects discussed above, lactate can also induce signaling through the G protein coupled receptor 132 (GPR132) in TAMs (Figure 2) [64]. Triggering of GPR132 by lactate induces a pro-tumorigenic TAM phenotype, characterized by expression of *Arg1*, promoting TAM recruitment and metastasis in vivo. In patients, GPR132 expression is associated with pro-tumoral TAM presence, breast cancer metastasis and poor prognosis [64]. Another study shows that pro-tumorigenic lactate signaling is mediated through extracellular signal-regulated kinase (ERK)/signal transducer and activator of transcription 3 (STAT3), inhibition of which decreases TAM presence in the TME and decreases tumor size and angiogenesis in breast cancer in vivo [65]. However, it is unclear whether GPR132 signaling is mediated via the ERK/STAT3 pathway.

Overall, lactate acts as a signaling molecule, skewing macrophages to a pro-tumorigenic phenotype. The signaling mechanisms might depend on other microenvironmental factors, such as hypoxia or co-signaling molecules, and could be cancer type specific. Nevertheless, promoting angiogenesis and metastasis appear to be common signatures of lactate signaling in macrophages.

### 4.2. Succinate Induces Tumor-Promoting Cytokine Production in Macrophages

As explained above, the oncometabolite succinate can accumulate in the TME. Recently, cancer-derived succinate was found to recruit monocytes, promote pro-tumorigenic TAM polarization and metastasis of human lung cancer through activation of succinate receptor (SUCNR1/GPR91) signaling (Figure 2) [23]. This activation triggers phosphoinositide 3-kinase (PI3K) signaling via the macrophage-dominant p110δ and p110γ PI3K isoforms, which control the HIF1α/VEGF axis [66]. Blockade of this signaling pathway shifts the TAM phenotype from pro- to anti-tumoral in vivo, leading to decreased metastatic nodules (Figure 2) [66].

However, succinate can also accumulate in activated macrophages. Lipopolysaccharide (LPS)-activated macrophages exhibit increased glycolysis and a truncated TCA cycle, accompanied by an increase in succinate levels [17]. Succinate is traditionally seen as a pro-inflammatory metabolite due to increased IL-1β production mediated by HIF1α stabilization [67]. Yet, IL-1β is associated with tumorigenesis by a direct induction of EMT [8]. Additionally, an anti-inflammatory role of succinate on macrophages has been described recently, decreasing CD86, CD80 and inducible nitric oxide (iNOS) expression and secretion of IL-6, tumor necrosis factor (TNF) and NO independently of GPR91 [68]. However, whether this mechanism also plays a role in TAM generation in vivo remains to be established.

In summary, these results indicate tumor- and macrophage-derived succinate as a pivotal signaling molecule in macrophages, potentially affecting tumor progression.

### 4.3. D-2HG Suppresses the Immune Response and Reduces Immune Cell Infiltration

As mentioned before, D-2HG can accumulate due to mutations in the IDH1/2 genes and supports tumorigenesis. The L-enantiomer of 2HG, produced under hypoxic conditions [69] and after T cell receptor (TCR) triggering, supports CD8^+^ T cell survival and anti-tumor responses in a lymphoma model in vivo [70]. In contrast, D-2HG suppresses anti-tumor T cell activity by interfering with TCR signaling and polyamine biosynthesis in a mouse glioma model [71]. Immune inhibitory functions were also found in dendritic cells (DCs) [72] and microglia, where D-2HG inhibits activation by AMP-activated protein kinase (AMPK)-mediated downregulation of mTOR and downstream nuclear factor κB (NF-κB)-induced inflammatory responses [73]. Interestingly, IDH mutations are generally associated with longer patient survival and better response to therapy [74], likely due to reduced myeloid cell infiltration and improved vasculature [75,76].

While it is clear that D-2HG affects immune responses and metabolism [71,73], additional research is required specifically into its effects on TAM polarization and function.

### 4.4. Kynurenine and Other Tryptophan Metabolites Inhibit the Anti-Tumor Response in TAMs

Increased expression of tryptophan (Trp) metabolism enzymes is often found in human brain, gynecological, kidney and lung cancers and has been associated with tumor growth and metastasis [43,44]. Kynurenine is a metabolite produced in the major metabolism pathway of Trp, which can be catalyzed by three enzymes: indoleamine 2,3-dioxygenase 1 (IDO1), IDO2 or tryptophan-2,3-dioxygenase (TDO). Multiple human cancer types such as brain and colon cancer secrete kynurenine into the TME [44,77], affecting macrophages in a paracrine manner (Figure 2) [78]. Kynurenine can signal through GPR35, which is highly expressed on macrophages [79]. Increased IDO and TDO expression in human melanoma is associated with an elevated abundance of TAMs with increased CD206 expression and decreased expression of *Nos2* (the gene encoding iNOS), *Il12, Tnf, Cd86* and *Cd40*. These TAMs also upregulate the kynurenine receptor aryl hydrocarbon receptor (AHR), inhibit CD8^+^ cytotoxic functions and engage in a pro-tumoral cooperation with Tregs [78]. Furthermore, blockade of AHR signaling enhances anti-programmed cell death protein 1 (αPD-1) therapy effects in a myeloid-dependent manner in a B16 melanoma model [78]. Additionally, kynurenine-activated AHR signaling was found to elevate C-C chemokine receptor type 2 (CCR2) expression, leading to monocyte recruitment and increased tumor growth in vivo [80]. AHR activation also decreases pro-inflammatory NF-κB signaling in TAMs and drives CD39 expression, leading to inhibition of CD8^+^ T cell function. High AHR expression in human gliomas is further associated with poor prognosis [80].

Other tryptophan metabolites, such as 3-hydroxykynurenine (3-HK), 3-hydroxyanthranilic acid (3-HAA) and quinolinic acid have also been implied to affect the immune response against cancer [81]. These molecules are consecutively produced after kynurenine catalyzation and can accumulate due to increased TDO or IDO1/2 activity. While the effects of these tryptophan metabolites on TAMs have not yet been elucidated, 3-HAA has been shown to inhibit NF-κB and inducible nitric oxide (iNOS) activation in macrophages [82].

Taken together, this research indicates a role for tryptophan metabolism and its metabolites in the communication between tumor cells and TAMs. Potential ways to exploit tryptophan metabolism as a therapeutic target are described in a later section. 

### 4.5. Production of Retinoic Acid by Tumor Cells Induces TAM Differentiation

Cancer cells have also been shown to metabolize vitamin A at elevated rates. Increased expression of aldehyde dehydrogenase 1a (ALHD1A), catalyzing the reaction of retinol to retinoic acid (RA), is correlated to poor cancer prognosis. While ALHD1A is used as a marker for malignancy and therapy response in different human cancer types including prostate and breast cancer [83,84], RA was considered an anti-tumor agent since it induces G1 arrest in breast cancer cells and thereby inhibits cell cycle progression [85], but its function is currently being revisited. RA has shown contradicting effects on immune cells, dependent on the microenvironment. Recently, it was shown in vivo that T cell-derived IL-13 promotes RA production by sarcoma cells, which causes differentiation of monocytes in the tumor into cancer-promoting TAMs rather than anti-tumor DCs [86]. Additionally, RA is associated with an immunosuppressive gene signature in human sarcoma, breast, lung and colon cancers and RA inhibition increases αPD-1 sensitivity in vivo, creating new prospects to enhance the effectiveness of immune checkpoint blockade therapy [86]. However, RA has also been found to decrease the ability of monocytes to respond to chemotactic stimuli, decreasing their recruitment to the tumor. Furthermore, RA treatment decreases macrophage IL-8 and VEGF production in vitro [87]. This discrepancy is likely due to different models used (i.e., in vivo vs. in vitro) and might also depend on cancer-type specificity of results.

In conclusion, the role of RA in cancer is currently under discussion with recent evidence indicating pro-tumoral properties through the creation of tumor-promoting macrophages. However, to elucidate the precise role of RA, additional research is required regarding cancer type specificity and in vivo mechanisms of TAM polarization.

## 5. Tumor-Derived Exosomes as Transporters of Oncometabolites

A potential mechanism for metabolic crosstalk between tumor cells and macrophages is the use of exosomes (Figure 1). Exosomes are microvesicles of around 50–150 nm and can be secreted by many cell types into the extracellular space, where they function as autocrine and paracrine communicators by carrying a variety of molecules including microRNA (miR), proteins, lipids, metabolites and metabolic enzymes [88,89].

Exosome release is increased in transformed cells, potentially induced by the conditions in the TME including acidity, hypoxia, and low nutrient concentrations. Exosomes have also been linked to tumor progression in sarcoma patients [90]. Exosome release, as well as uptake, is increased at low pH [91]. Furthermore, uptake efficiency in melanoma cells is increased for exosomes derived from metastatic cells compared to exosomes derived from primary melanoma cells, which may contribute to their metastatic capacity. Additionally, low pH and increased exosome release are linked to melanoma progression [92]. Recipient cells can take up exosomes through various mechanisms including endocytosis and fusion [93]. Macrophages can also internalize exosomes by PI3K-dependent phagocytosis [94]. Altogether, exosome release and uptake by macrophages is likely in cancer, suggesting a mechanism for immune evasion.

Indeed, an extensive body of evidence exists showing effects of tumor-derived exosomes on TAMs, promoting tumor progression [90,95] and immunosuppression [96,97] in human melanoma, leukemia, ovarian and esophageal tumors. Interestingly, tumor-derived exosomes have also been reported to induce metabolic changes in TAMs (Figure 1). A recent study indicates generation of pro-tumorigenic TAMs due to interference with lysosomal acid lipase A by miR-125b-5p shipped in melanoma-derived exosomes. Macrophages treated with these exosomes exhibit decreased expression of genes involved in lipid catabolism, reducing anti-tumor TAM functions [98]. Additionally, murine melanoma exosomes formed in hypoxia promote CD206^+^ TAMs together with a metabolic shift mediated by lethal-7a (let-7a) microRNA [99]. Hypoxic exosomes promote OXPHOS and increase REDD1 levels, subsequently suppressing the mTOR pathway in macrophages in vitro. Macrophages cultured with hypoxic exosomes increase B16 melanoma proliferation [99]. Breast cancer-derived exosomes can also prepare the premetastatic niche by suppressing glucose uptake and glycolysis of stromal cells in vivo by downregulation of glucose transporter 1 (GLUT1) and pyruvate kinase M2 (PKM2), leading to increased nutrient availability [100]. Additionally, hepatocellular carcinoma cell lines with a migratory phenotype encapsulate more sugar metabolism-associated proteins in exosomes than their non-migratory counterpart, such as the glycolysis enzyme glyceraldehyde 3-phosphate dehydrogenase (GAPDH) and proteins involved in glycolysis, gluconeogenesis, and the pentose phosphate pathway [101]. While exosome cargo has been reported to include metabolic enzymes and metabolites such as lactate, glutamic acid, amino acids, lipids, lactate dehydrogenase A and TCA cycle intermediates [88,102], most research about exosomes containing metabolites is focused on cancer-associated fibroblast (CAF)-derived exosomes changing cancer cell metabolism. Additional research is required to understand whether cancer cells employ exosome-mediated transfer of metabolic components to target TAM function.

Taken together, we suggest tumor-derived exosomes as a potential mode of metabolic crosstalk between cancer cells and TAMs. The extent to which exosomes influence TAM polarization in cancer patients and potential ways to exploit exosome transport for therapeutic targeting require further investigation.

## 6. Effect of TAM Metabolism on Tumor Progression

In addition to the more indirect effect of TAM metabolism on tumor progression via modulating macrophage effector functions, TAM metabolism affects tumorigenesis directly as well.

Tumor cells have been described to alter stromal cell metabolism in order to produce and secrete amino acids and biosynthetic building blocks to “feed” the cancer cells, including amino acids, lipids and TCA cycle intermediates [88,103,104,105]. While the main mechanism by which TAMs feed tumor cells is angiogenesis, TAMs may engage in similar production and secretion of nutrients. Recently, thyroid carcinoma- and neuroblastoma-induced macrophages were found to upregulate lipid biosynthesis in vitro, supporting their pro-tumoral function including cytokine production [106]. Combined with the finding that macrophages can secrete lipids in vitro and in vivo [107,108], and TAM-derived extracellular vesicles containing lipids [89] this may indicate that not only CAFs and adipocytes but also TAMs “feed” cancer cells. Additionally, Arg1-mediated polyamine production by macrophages increases breast cancer cell proliferation in vitro [109]. However, whether this indeed occurs in the in vivo TME requires further investigation.

TAMs can also directly alter tumor cell metabolism by secreting cytokines bearing metabolic effects. A glycolytic phenotype of cancer cells is associated with worse prognosis and stimulated by TAM-derived TNF [110,111], IL-6 [111], C-C motif chemokine ligand 8 (CCL8) [112] and HIF1α-stabilizing long noncoding RNA transported in extracellular vesicles [113] in different human cancers including glioblastoma, lung and breast cancer. Specifically, lactate-activated macrophages produce CCL5, which increases cell migration, induces EMT, and promotes aerobic glycolysis in an in vivo breast cancer model [114]. Furthermore, effects of TAM metabolism have been reported on tumor vasculature and metastasis as increasing TAM glycolysis results in glucose competition with endothelial cells, stabilizing the microvasculature [37]. Interestingly, pyrimidine secretion by pro-tumoral TAMs has been shown to worsen the in vivo response to chemotherapy through competition [115].

Although these results are intriguing and might provide targets for therapy, direct effects of TAM metabolism on tumor metabolism and progression have not been extensively studied and additional research is required.

## 7. Future Perspectives

Metabolic interactions are being recognized as a crucial aspect for cancer cell survival and immune evasion. Tumor cells do not just utilize one form of communication, but rather multiple factors to increase their chances of survival, including many that are not (directly) related to metabolism.

Since macrophage metabolism and function are intertwined, TAM metabolism is an interesting topic in cancer research, specifically as their plasticity may allow for metabolic reprogramming towards anti-tumor responses. However, very few studies directly measure TAM metabolism in an in vivo setting [37] and most research has either been conducted on in vitro TAM models or assesses only gene expression rather than metabolic flux [22,34,35,36]. This may also lead to conflicting results, such as differential regulation of glycolysis in TAMs; while glycolysis is found to be inhibited [37,38], other studies indicate upregulation of glycolytic enzymes [34,35,36]. However, TAM metabolism and function may also differ per cancer (sub)type or in regard to spatiotemporal characteristics of the TME. Furthermore, it is often unclear whether altered TAM metabolism is directly tumor-induced or simply a compensatory mechanism, indicating the need for further research in this area.

Reversing (metabolic) polarization towards pro-tumoral TAMs or re-educating TAMs to gain anti-tumor responses could decrease metastasis and enhance immune effector functions [37,41,65,66,78,86,116]. Oncometabolites can induce cancer-promoting signaling pathways in macrophages, including PI3K signaling via the protein kinase B (PKB/AKT) and mTOR pathways. Targeted blockade of PI3K repolarizes pro- to anti-tumoral TAMs and restores CD8^+^ T cell effector functions in vivo [117,118]. Oncometabolites themselves are also being investigated as therapy targets [119,120]. Interfering with other metabolic pathways in TAMs also yields promising preclinical data that may ultimately be translated into new avenues for cancer therapy. For example, lipid metabolism and fatty acid oxidation have been identified as upregulated in TAMs and involved in pro-tumoral polarization, thereby presenting promising therapy targets. TAMs are found to accumulate lipids as an alternative fuel source in vitro and inhibiting this mechanism leads to reduction in tumor growth in vivo [121,122]. Additionally, targeting cholesterol metabolism elicits anti-tumor functions in TAMs and decreases EMT in vivo, improving chemotherapy resistance [123]. Another encouraging example is presented by inhibiting glutamine metabolism of both cancer and myeloid cells, which leads to increased frequencies of anti-tumoral TAMs and reduced tumor growth. Unexpectedly, inhibiting glutamine metabolism simultaneously suppresses IDO expression in both tumor and myeloid cells, decreasing kynurenine levels and further promoting tumoricidal activity. This also decreases resistance against immunotherapy [124]. Tryptophan metabolism constitutes a promising metabolic target in general, as IDO/TDO inhibitors may decrease tumor kynurenine production while simultaneously providing tryptophan to T cells in the TME, enabling them to carry out anti-tumor responses [125,126]. Decreased kynurenine levels or AHR blockade may additionally counteract pro-tumoral TAM polarization and improve αPD-1 therapy response [78,125,126]. However, although promising in earlier clinical trials, in a phase III trial with an IDO inhibitor, the latter effect could not be replicated in metastatic melanoma patients [127]. This indicates the need to validate results obtained through mouse models in patients, as results in mice cannot be directly translated to human disease. Nevertheless, targeting tryptophan metabolism at other levels than IDO expression, such as AHR blockade, remains under investigation as an extensive body of preclinical evidence implicates this pathway in tumor progression and decreased response to immune therapy [127]. Taken together, targeting TAM metabolism is a promising avenue for cancer therapy.

However, some challenges remain. One of these is specific targeting of macrophages, as cancer cells and immune effector cells show a similar metabolic profile with increased reliance on glycolysis. Therefore, targeting cancer glycolysis or glucose availability may have adverse effects as tumoricidal TAMs and CD8^+^ effector cells may also be hindered in their function [37,128]. Therefore, specific macrophage receptors or genes are preferred for targeting macrophages, or finding targets that have beneficial effects regardless of targeting cancer or myeloid cells, such as shown for glutamine [124]. Nevertheless, if immune cell subsets are metabolically sufficiently different from cancer cells, this may also provide targeting opportunities, as further discussed here [125,126].

The increasing knowledge regarding macrophage and cancer cell metabolism contributes to our understanding of communication in the TME. However, more research is needed into the precise signaling mechanisms of metabolic crosstalk between cancer cells and macrophages for the development of novel cancer therapies.

## References

[1] Hanahan D., Weinberg R.A. (2011). Hallmarks of cancer: The next generation. Cell.

[2] Kelly P.M., Davison R.S., Bliss E., McGee J.O. (1988). Macrophages in human breast disease: A quantitative immunohistochemical study. Br. J. Cancer.

[3] Zhang Q.-W., Liu L., Gong C.-Y., Shi H.-S., Zeng Y.-H., Wang X.-Z., Zhao Y.-W., Wei Y.-Q. (2012). Prognostic Significance of Tumor-Associated Macrophages in Solid Tumor: A Meta-Analysis of the Literature. PLoS ONE.

[4] Gentles A.J., Newman A.M., Liu C.L., Bratman S.V., Feng W., Kim D., Nair V.S., Xu Y., Khuong A., Hoang C.D. (2015). The prognostic landscape of genes and infiltrating immune cells across human cancers. Nat. Med..

[5] Bolli E., Movahedi K., Laoui D., Van Ginderachter J.A. (2017). Novel insights in the regulation and function of macrophages in the tumor microenvironment. Curr. Opin. Oncol..

[6] Carmona-Fontaine C., Deforet M., Akkari L., Thompson C.B., Joyce J.A., Xavier J.B. (2017). Metabolic origins of spatial organization in the tumor microenvironment. Proc. Natl. Acad. Sci. USA.

[7] Movahedi K., Laoui D., Gysemans C., Baeten M., Stange G., Van den Bossche J., Mack M., Pipeleers D., In’t Veld P., De Baetselier P. (2010). Different tumor microenvironments contain functionally distinct subsets of macrophages derived from Ly6C(high) monocytes. Cancer Res..

[8] Greten F.R., Grivennikov S.I. (2019). Inflammation and Cancer: Triggers, Mechanisms, and Consequences. Immunity.

[9] Honkanen T.J., Tikkanen A., Karihtala P., Makinen M., Vayrynen J.P., Koivunen J.P. (2019). Prognostic and predictive role of tumour-associated macrophages in HER2 positive breast cancer. Sci. Rep..

[10] Noman M.Z., Desantis G., Janji B., Hasmim M., Karray S., Dessen P., Bronte V., Chouaib S. (2014). PD-L1 is a novel direct target of HIF-1alpha, and its blockade under hypoxia enhanced MDSC-mediated T cell activation. J. Exp. Med..

[11] Sica A., Saccani A., Bottazzi B., Polentarutti N., Vecchi A., van Damme J., Mantovani A. (2000). Autocrine production of IL-10 mediates defective IL-12 production and NF-kappa B activation in tumor-associated macrophages. J. Immunol..

[12] Su S., Liao J., Liu J., Huang D., He C., Chen F., Yang L., Wu W., Chen J., Lin L. (2017). Blocking the recruitment of naive CD4(+) T cells reverses immunosuppression in breast cancer. Cell Res..

[13] Zhou J., Li X., Wu X., Zhang T., Zhu Q., Wang X., Wang H., Wang K., Lin Y., Wang X. (2018). Exosomes Released from Tumor-Associated Macrophages Transfer miRNAs That Induce a Treg/Th17 Cell Imbalance in Epithelial Ovarian Cancer. Cancer Immunol. Res..

[14] Shalapour S., Karin M. (2019). Pas de Deux: Control of Anti-tumor Immunity by Cancer-Associated Inflammation. Immunity.

[15] Van den Bossche J., O’Neill L.A., Menon D. (2017). Macrophage Immunometabolism: Where Are We (Going)?. Trends Immunol..

[16] Jha A.K., Huang S.C., Sergushichev A., Lampropoulou V., Ivanova Y., Loginicheva E., Chmielewski K., Stewart K.M., Ashall J., Everts B. (2015). Network integration of parallel metabolic and transcriptional data reveals metabolic modules that regulate macrophage polarization. Immunity.

[17] Mills E.L., Kelly B., Logan A., Costa A.S.H., Varma M., Bryant C.E., Tourlomousis P., Dabritz J.H.M., Gottlieb E., Latorre I. (2016). Succinate Dehydrogenase Supports Metabolic Repurposing of Mitochondria to Drive Inflammatory Macrophages. Cell.

[18] Huang S.C., Everts B., Ivanova Y., O’Sullivan D., Nascimento M., Smith A.M., Beatty W., Love-Gregory L., Lam W.Y., O’Neill C.M. (2014). Cell-intrinsic lysosomal lipolysis is essential for alternative activation of macrophages. Nat. Immunol..

[19] Huang S.C., Smith A.M., Everts B., Colonna M., Pearce E.L., Schilling J.D., Pearce E.J. (2016). Metabolic Reprogramming Mediated by the mTORC2-IRF4 Signaling Axis Is Essential for Macrophage Alternative Activation. Immunity.

[20] Warburg O., Wind F., Negelein E. (1927). The Metabolism of Tumors in the Body. J. Gen. Physiol..

[21] DeBerardinis R.J., Chandel N.S. (2020). We need to talk about the Warburg effect. Nat. Metab..

[22] Colegio O.R., Chu N.Q., Szabo A.L., Chu T., Rhebergen A.M., Jairam V., Cyrus N., Brokowski C.E., Eisenbarth S.C., Phillips G.M. (2014). Functional polarization of tumour-associated macrophages by tumour-derived lactic acid. Nature.

[23] Wu J.Y., Huang T.W., Hsieh Y.T., Wang Y.F., Yen C.C., Lee G.L., Yeh C.C., Peng Y.J., Kuo Y.Y., Wen H.T. (2020). Cancer-Derived Succinate Promotes Macrophage Polarization and Cancer Metastasis via Succinate Receptor. Mol. Cell.

[24] Dang L., White D.W., Gross S., Bennett B.D., Bittinger M.A., Driggers E.M., Fantin V.R., Jang H.G., Jin S., Keenan M.C. (2009). Cancer-associated IDH1 mutations produce 2-hydroxyglutarate. Nature.

[25] Jain R.K. (2005). Normalization of tumor vasculature: An emerging concept in antiangiogenic therapy. Science.

[26] Rofstad E.K., Gaustad J.V., Egeland T.A., Mathiesen B., Galappathi K. (2010). Tumors exposed to acute cyclic hypoxic stress show enhanced angiogenesis, perfusion and metastatic dissemination. Int. J. Cancer.

[27] Kim J.W., Tchernyshyov I., Semenza G.L., Dang C.V. (2006). HIF-1-mediated expression of pyruvate dehydrogenase kinase: A metabolic switch required for cellular adaptation to hypoxia. Cell Metab..

[28] Fang H.Y., Hughes R., Murdoch C., Coffelt S.B., Biswas S.K., Harris A.L., Johnson R.S., Imityaz H.Z., Simon M.C., Fredlund E. (2009). Hypoxia-inducible factors 1 and 2 are important transcriptional effectors in primary macrophages experiencing hypoxia. Blood.

[29] Sonveaux P., Vegran F., Schroeder T., Wergin M.C., Verrax J., Rabbani Z.N., De Saedeleer C.J., Kennedy K.M., Diepart C., Jordan B.F. (2008). Targeting lactate-fueled respiration selectively kills hypoxic tumor cells in mice. J. Clin. Invest..

[30] Bohn T., Rapp S., Luther N., Klein M., Bruehl T.J., Kojima N., Aranda Lopez P., Hahlbrock J., Muth S., Endo S. (2018). Tumor immunoevasion via acidosis-dependent induction of regulatory tumor-associated macrophages. Nat. Immunol..

[31] Tripathi C., Tewari B.N., Kanchan R.K., Baghel K.S., Nautiyal N., Shrivastava R., Kaur H., Bhatt M.L., Bhadauria S. (2014). Macrophages are recruited to hypoxic tumor areas and acquire a pro-angiogenic M2-polarized phenotype via hypoxic cancer cell derived cytokines Oncostatin M and Eotaxin. Oncotarget.

[32] Hirayama A., Kami K., Sugimoto M., Sugawara M., Toki N., Onozuka H., Kinoshita T., Saito N., Ochiai A., Tomita M. (2009). Quantitative metabolome profiling of colon and stomach cancer microenvironment by capillary electrophoresis time-of-flight mass spectrometry. Cancer Res..

[33] Ho P.C., Bihuniak J.D., Macintyre A.N., Staron M., Liu X., Amezquita R., Tsui Y.C., Cui G., Micevic G., Perales J.C. (2015). Phosphoenolpyruvate Is a Metabolic Checkpoint of Anti-tumor T Cell Responses. Cell.

[34] Liu D., Chang C., Lu N., Wang X., Lu Q., Ren X., Ren P., Zhao D., Wang L., Zhu Y. (2017). Comprehensive Proteomics Analysis Reveals Metabolic Reprogramming of Tumor-Associated Macrophages Stimulated by the Tumor Microenvironment. J. Proteome Res..

[35] Penny H.L., Sieow J.L., Adriani G., Yeap W.H., See Chi Ee P., San Luis B., Lee B., Lee T., Mak S.Y., Ho Y.S. (2016). Warburg metabolism in tumor-conditioned macrophages promotes metastasis in human pancreatic ductal adenocarcinoma. OncoImmunology.

[36] Arts R.J., Plantinga T.S., Tuit S., Ulas T., Heinhuis B., Tesselaar M., Sloot Y., Adema G.J., Joosten L.A., Smit J.W. (2016). Transcriptional and metabolic reprogramming induce an inflammatory phenotype in non-medullary thyroid carcinoma-induced macrophages. OncoImmunology.

[37] Wenes M., Shang M., Di Matteo M., Goveia J., Martin-Perez R., Serneels J., Prenen H., Ghesquiere B., Carmeliet P., Mazzone M. (2016). Macrophage Metabolism Controls Tumor Blood Vessel Morphogenesis and Metastasis. Cell Metab..

[38] Zhihua Y., Yulin T., Yibo W., Wei D., Yin C., Jiahao X., Runqiu J., Xuezhong X. (2019). Hypoxia decreases macrophage glycolysis and M1 percentage by targeting microRNA-30c and mTOR in human gastric cancer. Cancer Sci..

[39] Zhou R., Pantel A.R., Li S., Lieberman B.P., Ploessl K., Choi H., Blankemeyer E., Lee H., Kung H.F., Mach R.H. (2017). [(18)F](2S,4R)4-Fluoroglutamine PET Detects Glutamine Pool Size Changes in Triple-Negative Breast Cancer in Response to Glutaminase Inhibition. Cancer Res..

[40] Choi J., Stradmann-Bellinghausen B., Yakubov E., Savaskan N.E., Regnier-Vigouroux A. (2015). Glioblastoma cells induce differential glutamatergic gene expressions in human tumor-associated microglia/macrophages and monocyte-derived macrophages. Cancer Biol. Ther..

[41] Palmieri E.M., Menga A., Martin-Perez R., Quinto A., Riera-Domingo C., De Tullio G., Hooper D.C., Lamers W.H., Ghesquiere B., McVicar D.W. (2017). Pharmacologic or Genetic Targeting of Glutamine Synthetase Skews Macrophages toward an M1-like Phenotype and Inhibits Tumor Metastasis. Cell Rep..

[42] Kostourou V., Cartwright J.E., Johnstone A.P., Boult J.K., Cullis E.R., Whitley G., Robinson S.P. (2011). The role of tumour-derived iNOS in tumour progression and angiogenesis. Br. J. Cancer.

[43] Theate I., van Baren N., Pilotte L., Moulin P., Larrieu P., Renauld J.C., Herve C., Gutierrez-Roelens I., Marbaix E., Sempoux C. (2015). Extensive profiling of the expression of the indoleamine 2,3-dioxygenase 1 protein in normal and tumoral human tissues. Cancer Immunol. Res..

[44] Opitz C.A., Litzenburger U.M., Sahm F., Ott M., Tritschler I., Trump S., Schumacher T., Jestaedt L., Schrenk D., Weller M. (2011). An endogenous tumour-promoting ligand of the human aryl hydrocarbon receptor. Nature.

[45] Rodriguez P.C., Zea A.H., DeSalvo J., Culotta K.S., Zabaleta J., Quiceno D.G., Ochoa J.B., Ochoa A.C. (2003). L-arginine consumption by macrophages modulates the expression of CD3 zeta chain in T lymphocytes. J. Immunol..

[46] Geiger R., Rieckmann J.C., Wolf T., Basso C., Feng Y., Fuhrer T., Kogadeeva M., Picotti P., Meissner F., Mann M. (2016). L-Arginine Modulates T Cell Metabolism and Enhances Survival and Anti-tumor Activity. Cell.

[47] Fallarino F., Grohmann U., You S., McGrath B.C., Cavener D.R., Vacca C., Orabona C., Bianchi R., Belladonna M.L., Volpi C. (2006). The combined effects of tryptophan starvation and tryptophan catabolites down-regulate T cell receptor zeta-chain and induce a regulatory phenotype in naive T cells. J. Immunol..

[48] Tan A.S., Baty J.W., Dong L.F., Bezawork-Geleta A., Endaya B., Goodwin J., Bajzikova M., Kovarova J., Peterka M., Yan B. (2015). Mitochondrial genome acquisition restores respiratory function and tumorigenic potential of cancer cells without mitochondrial DNA. Cell Metab..

[49] LeBleu V.S., O’Connell J.T., Gonzalez Herrera K.N., Wikman H., Pantel K., Haigis M.C., de Carvalho F.M., Damascena A., Domingos Chinen L.T., Rocha R.M. (2014). PGC-1alpha mediates mitochondrial biogenesis and oxidative phosphorylation in cancer cells to promote metastasis. Nat. Cell Biol..

[50] Sciacovelli M., Frezza C. (2016). Oncometabolites: Unconventional triggers of oncogenic signalling cascades. Free Radic Biol. Med..

[51] Pollard P.J., Briere J.J., Alam N.A., Barwell J., Barclay E., Wortham N.C., Hunt T., Mitchell M., Olpin S., Moat S.J. (2005). Accumulation of Krebs cycle intermediates and over-expression of HIF1alpha in tumours which result from germline FH and SDH mutations. Hum. Mol. Genet..

[52] Burnichon N., Briere J.J., Libe R., Vescovo L., Riviere J., Tissier F., Jouanno E., Jeunemaitre X., Benit P., Tzagoloff A. (2010). SDHA is a tumor suppressor gene causing paraganglioma. Hum. Mol. Genet..

[53] Gimenez-Roqueplo A.P., Favier J., Rustin P., Rieubland C., Crespin M., Nau V., Khau Van Kien P., Corvol P., Plouin P.F., Jeunemaitre X. (2003). Mutations in the SDHB gene are associated with extra-adrenal and/or malignant phaeochromocytomas. Cancer Res..

[54] Bourdeau I., Grunenwald S., Burnichon N., Khalifa E., Dumas N., Binet M.C., Nolet S., Gimenez-Roqueplo A.P. (2016). A SDHC Founder Mutation Causes Paragangliomas (PGLs) in the French Canadians: New Insights on the SDHC-Related PGL. J. Clin. Endocrinol. Metab..

[55] Habano W., Sugai T., Nakamura S., Uesugi N., Higuchi T., Terashima M., Horiuchi S. (2003). Reduced expression and loss of heterozygosity of the SDHD gene in colorectal and gastric cancer. Oncol. Rep..

[56] Losman J.A., Looper R.E., Koivunen P., Lee S., Schneider R.K., McMahon C., Cowley G.S., Root D.E., Ebert B.L., Kaelin W.G. (2013). (R)-2-hydroxyglutarate is sufficient to promote leukemogenesis and its effects are reversible. Science.

[57] Letouze E., Martinelli C., Loriot C., Burnichon N., Abermil N., Ottolenghi C., Janin M., Menara M., Nguyen A.T., Benit P. (2013). SDH mutations establish a hypermethylator phenotype in paraganglioma. Cancer Cell.

[58] Miranda-Goncalves V., Lameirinhas A., Macedo-Silva C., Lobo J., C Dias P., Ferreira V., Henrique R., Jeronimo C. (2020). Lactate Increases Renal Cell Carcinoma Aggressiveness through Sirtuin 1-Dependent Epithelial Mesenchymal Transition Axis Regulation. Cells.

[59] Aspuria P.P., Lunt S.Y., Varemo L., Vergnes L., Gozo M., Beach J.A., Salumbides B., Reue K., Wiedemeyer W.R., Nielsen J. (2014). Succinate dehydrogenase inhibition leads to epithelial-mesenchymal transition and reprogrammed carbon metabolism. Cancer Metab..

[60] Sciacovelli M., Goncalves E., Johnson T.I., Zecchini V.R., da Costa A.S., Gaude E., Drubbel A.V., Theobald S.J., Abbo S.R., Tran M.G. (2016). Fumarate is an epigenetic modifier that elicits epithelial-to-mesenchymal transition. Nature.

[61] Colvin H., Nishida N., Konno M., Haraguchi N., Takahashi H., Nishimura J., Hata T., Kawamoto K., Asai A., Tsunekuni K. (2016). Oncometabolite D-2-Hydroxyglurate Directly Induces Epithelial-Mesenchymal Transition and is Associated with Distant Metastasis in Colorectal Cancer. Sci. Rep..

[62] Delahousse J., Verlingue L., Broutin S., Legoupil C., Touat M., Doucet L., Ammari S., Lacroix L., Ducreux M., Scoazec J.Y. (2018). Circulating oncometabolite D-2-hydroxyglutarate enantiomer is a surrogate marker of isocitrate dehydrogenase-mutated intrahepatic cholangiocarcinomas. Eur. J. Cancer.

[63] Zhang D., Tang Z., Huang H., Zhou G., Cui C., Weng Y., Liu W., Kim S., Lee S., Perez-Neut M. (2019). Metabolic regulation of gene expression by histone lactylation. Nature.

[64] Chen P., Zuo H., Xiong H., Kolar M.J., Chu Q., Saghatelian A., Siegwart D.J., Wan Y. (2017). Gpr132 sensing of lactate mediates tumor-macrophage interplay to promote breast cancer metastasis. Proc. Natl. Acad. Sci. USA.

[65] Mu X., Shi W., Xu Y., Xu C., Zhao T., Geng B., Yang J., Pan J., Hu S., Zhang C. (2018). Tumor-derived lactate induces M2 macrophage polarization via the activation of the ERK/STAT3 signaling pathway in breast cancer. Cell Cycle.

[66] Joshi S., Singh A.R., Zulcic M., Durden D.L. (2014). A macrophage-dominant PI3K isoform controls hypoxia-induced HIF1alpha and HIF2alpha stability and tumor growth, angiogenesis, and metastasis. Mol. Cancer Res..

[67] Tannahill G.M., Curtis A.M., Adamik J., Palsson-McDermott E.M., McGettrick A.F., Goel G., Frezza C., Bernard N.J., Kelly B., Foley N.H. (2013). Succinate is an inflammatory signal that induces IL-1beta through HIF-1alpha. Nature.

[68] Harber K.J., de Goede K.E., Verberk S.G.S., Meinster E., de Vries H.E., van Weeghel M., de Winther M.P.J., Van den Bossche J. (2020). Succinate Is an Inflammation-Induced Immunoregulatory Metabolite in Macrophages. Metabolites.

[69] Intlekofer A.M., Dematteo R.G., Venneti S., Finley L.W., Lu C., Judkins A.R., Rustenburg A.S., Grinaway P.B., Chodera J.D., Cross J.R. (2015). Hypoxia Induces Production of L-2-Hydroxyglutarate. Cell Metab..

[70] Tyrakis P.A., Palazon A., Macias D., Lee K.L., Phan A.T., Velica P., You J., Chia G.S., Sim J., Doedens A. (2016). S-2-hydroxyglutarate regulates CD8(+) T-lymphocyte fate. Nature.

[71] Bunse L., Pusch S., Bunse T., Sahm F., Sanghvi K., Friedrich M., Alansary D., Sonner J.K., Green E., Deumelandt K. (2018). Suppression of antitumor T cell immunity by the oncometabolite (R)-2-hydroxyglutarate. Nat. Med..

[72] Ugele I., Cardenas-Conejo Z.E., Hammon K., Wehrstein M., Bruss C., Peter K., Singer K., Gottfried E., Boesch J., Oefner P. (2019). D-2-Hydroxyglutarate and L-2-Hydroxyglutarate Inhibit IL-12 Secretion by Human Monocyte-Derived Dendritic Cells. Int. J. Mol. Sci..

[73] Han C.J., Zheng J.Y., Sun L., Yang H.C., Cao Z.Q., Zhang X.H., Zheng L.T., Zhen X.C. (2019). The oncometabolite 2-hydroxyglutarate inhibits microglial activation via the AMPK/mTOR/NF-kappaB pathway. Acta Pharmacol. Sin..

[74] SongTao Q., Lei Y., Si G., YanQing D., HuiXia H., XueLin Z., LanXiao W., Fei Y. (2012). IDH mutations predict longer survival and response to temozolomide in secondary glioblastoma. Cancer Sci..

[75] Zhang L., He L., Lugano R., Roodakker K., Bergqvist M., Smits A., Dimberg A. (2018). IDH mutation status is associated with distinct vascular gene expression signatures in lower-grade gliomas. Neuro Oncol..

[76] Amankulor N.M., Kim Y., Arora S., Kargl J., Szulzewsky F., Hanke M., Margineantu D.H., Rao A., Bolouri H., Delrow J. (2017). Mutant IDH1 regulates the tumor-associated immune system in gliomas. Genes Dev..

[77] Venkateswaran N., Lafita-Navarro M.C., Hao Y.H., Kilgore J.A., Perez-Castro L., Braverman J., Borenstein-Auerbach N., Kim M., Lesner N.P., Mishra P. (2019). MYC promotes tryptophan uptake and metabolism by the kynurenine pathway in colon cancer. Genes Dev..

[78] Campesato L.F., Budhu S., Tchaicha J., Weng C.H., Gigoux M., Cohen I.J., Redmond D., Mangarin L., Pourpe S., Liu C. (2020). Blockade of the AHR restricts a Treg-macrophage suppressive axis induced by L-Kynurenine. Nat. Commun..

[79] Wang J., Simonavicius N., Wu X., Swaminath G., Reagan J., Tian H., Ling L. (2006). Kynurenic acid as a ligand for orphan G protein-coupled receptor GPR35. J. Biol. Chem..

[80] Takenaka M.C., Gabriely G., Rothhammer V., Mascanfroni I.D., Wheeler M.A., Chao C.C., Gutierrez-Vazquez C., Kenison J., Tjon E.C., Barroso A. (2019). Control of tumor-associated macrophages and T cells in glioblastoma via AHR and CD39. Nat. NeuroSci..

[81] Yu C.P., Song Y.L., Zhu Z.M., Huang B., Xiao Y.Q., Luo D.Y. (2017). Targeting TDO in cancer immunotherapy. Med. Oncol..

[82] Sekkai D., Guittet O., Lemaire G., Tenu J.P., Lepoivre M. (1997). Inhibition of nitric oxide synthase expression and activity in macrophages by 3-hydroxyanthranilic acid, a tryptophan metabolite. Arch. Biochem. Biophys..

[83] Li T., Su Y., Mei Y., Leng Q., Leng B., Liu Z., Stass S.A., Jiang F. (2010). ALDH1A1 is a marker for malignant prostate stem cells and predictor of prostate cancer patients’ outcome. Lab. Invest..

[84] Khoury T., Ademuyiwa F.O., Chandrasekhar R., Jabbour M., Deleo A., Ferrone S., Wang Y., Wang X. (2012). Aldehyde dehydrogenase 1A1 expression in breast cancer is associated with stage, triple negativity, and outcome to neoadjuvant chemotherapy. Mod. Pathol..

[85] Mangiarotti R., Danova M., Alberici R., Pellicciari C. (1998). All-trans retinoic acid (ATRA)-induced apoptosis is preceded by G1 arrest in human MCF-7 breast cancer cells. Br. J. Cancer.

[86] Devalaraja S., To T.K.J., Folkert I.W., Natesan R., Alam M.Z., Li M., Tada Y., Budagyan K., Dang M.T., Zhai L. (2020). Tumor-Derived Retinoic Acid Regulates Intratumoral Monocyte Differentiation to Promote Immune Suppression. Cell.

[87] Liss C., Fekete M.J., Hasina R., Lingen M.W. (2002). Retinoic acid modulates the ability of macrophages to participate in the induction of the angiogenic phenotype in head and neck squamous cell carcinoma. Int. J. Cancer.

[88] Zhao H., Yang L., Baddour J., Achreja A., Bernard V., Moss T., Marini J.C., Tudawe T., Seviour E.G., San Lucas F.A. (2016). Tumor microenvironment derived exosomes pleiotropically modulate cancer cell metabolism. eLife.

[89] Cianciaruso C., Beltraminelli T., Duval F., Nassiri S., Hamelin R., Mozes A., Gallart-Ayala H., Ceada Torres G., Torchia B., Ries C.H. (2019). Molecular Profiling and Functional Analysis of Macrophage-Derived Tumor Extracellular Vesicles. Cell Rep..

[90] Casadei L., Calore F., Creighton C.J., Guescini M., Batte K., Iwenofu O.H., Zewdu A., Braggio D.A., Bill K.L., Fadda P. (2017). Exosome-Derived miR-25-3p and miR-92a-3p Stimulate Liposarcoma Progression. Cancer Res..

[91] Parolini I., Federici C., Raggi C., Lugini L., Palleschi S., De Milito A., Coscia C., Iessi E., Logozzi M., Molinari A. (2009). Microenvironmental pH is a key factor for exosome traffic in tumor cells. J. Biol. Chem..

[92] Boussadia Z., Lamberti J., Mattei F., Pizzi E., Puglisi R., Zanetti C., Pasquini L., Fratini F., Fantozzi L., Felicetti F. (2018). Acidic microenvironment plays a key role in human melanoma progression through a sustained exosome mediated transfer of clinically relevant metastatic molecules. J. Exp. Clin. Cancer Res..

[93] Mathieu M., Martin-Jaular L., Lavieu G., Thery C. (2019). Specificities of secretion and uptake of exosomes and other extracellular vesicles for cell-to-cell communication. Nat. Cell Biol..

[94] Feng D., Zhao W.L., Ye Y.Y., Bai X.C., Liu R.Q., Chang L.F., Zhou Q., Sui S.F. (2010). Cellular internalization of exosomes occurs through phagocytosis. Traffic.

[95] Ying X., Wu Q., Wu X., Zhu Q., Wang X., Jiang L., Chen X., Wang X. (2016). Epithelial ovarian cancer-secreted exosomal miR-222-3p induces polarization of tumor-associated macrophages. Oncotarget.

[96] Haderk F., Schulz R., Iskar M., Cid L.L., Worst T., Willmund K.V., Schulz A., Warnken U., Seiler J., Benner A. (2017). Tumor-derived exosomes modulate PD-L1 expression in monocytes. Sci. Immunol..

[97] Li B., Song T.N., Wang F.R., Yin C., Li Z., Lin J.P., Meng Y.Q., Feng H.M., Jing T. (2019). Tumor-derived exosomal HMGB1 promotes esophageal squamous cell carcinoma progression through inducing PD1(+) TAM expansion. OncoGenesis.

[98] Gerloff D., Lutzkendorf J., Moritz R.K.C., Wersig T., Mader K., Muller L.P., Sunderkotter C. (2020). Melanoma-Derived Exosomal miR-125b-5p Educates Tumor Associated Macrophages (TAMs) by Targeting Lysosomal Acid Lipase A (LIPA). Cancers.

[99] Park J.E., Dutta B., Tse S.W., Gupta N., Tan C.F., Low J.K., Yeoh K.W., Kon O.L., Tam J.P., Sze S.K. (2019). Hypoxia-induced tumor exosomes promote M2-like macrophage polarization of infiltrating myeloid cells and microRNA-mediated metabolic shift. OncoGene.

[100] Fong M.Y., Zhou W., Liu L., Alontaga A.Y., Chandra M., Ashby J., Chow A., O’Connor S.T., Li S., Chin A.R. (2015). Breast-cancer-secreted miR-122 reprograms glucose metabolism in premetastatic niche to promote metastasis. Nat. Cell Biol..

[101] Zhang J., Lu S., Zhou Y., Meng K., Chen Z., Cui Y., Shi Y., Wang T., He Q.Y. (2017). Motile hepatocellular carcinoma cells preferentially secret sugar metabolism regulatory proteins via exosomes. Proteomics.

[102] Vallabhaneni K.C., Penfornis P., Dhule S., Guillonneau F., Adams K.V., Mo Y.Y., Xu R., Liu Y., Watabe K., Vemuri M.C. (2015). Extracellular vesicles from bone marrow mesenchymal stem/stromal cells transport tumor regulatory microRNA, proteins, and metabolites. Oncotarget.

[103] Yang L., Achreja A., Yeung T.L., Mangala L.S., Jiang D., Han C., Baddour J., Marini J.C., Ni J., Nakahara R. (2016). Targeting Stromal Glutamine Synthetase in Tumors Disrupts Tumor Microenvironment-Regulated Cancer Cell Growth. Cell Metab..

[104] Sousa C.M., Biancur D.E., Wang X., Halbrook C.J., Sherman M.H., Zhang L., Kremer D., Hwang R.F., Witkiewicz A.K., Ying H. (2016). Pancreatic stellate cells support tumour metabolism through autophagic alanine secretion. Nature.

[105] Nieman K.M., Kenny H.A., Penicka C.V., Ladanyi A., Buell-Gutbrod R., Zillhardt M.R., Romero I.L., Carey M.S., Mills G.B., Hotamisligil G.S. (2011). Adipocytes promote ovarian cancer metastasis and provide energy for rapid tumor growth. Nat. Med..

[106] Rabold K., Aschenbrenner A., Thiele C., Boahen C.K., Schiltmans A., Smit J.W.A., Schultze J.L., Netea M.G., Adema G.J., Netea-Maier R.T. (2020). Enhanced lipid biosynthesis in human tumor-induced macrophages contributes to their protumoral characteristics. J. Immunother. Cancer.

[107] Peres C.M., de Bittencourt P.I., da Costa M., Curi R. (1997). Transference of fatty acids from macrophages to lymphocytes in culture. Biochem. Soc. Trans..

[108] Peres C.M., Homem de Bittencourt P.I., Mendonca J.R., Curi R. (2003). Evidence that macrophages transfer arachidonic acid and cholesterol to tissues in vivo. Cell Biochem. Funct..

[109] Chang C.I., Liao J.C., Kuo L. (2001). Macrophage arginase promotes tumor cell growth and suppresses nitric oxide-mediated tumor cytotoxicity. Cancer Res..

[110] Jeong H., Kim S., Hong B.J., Lee C.J., Kim Y.E., Bok S., Oh J.M., Gwak S.H., Yoo M.Y., Lee M.S. (2019). Tumor-Associated Macrophages Enhance Tumor Hypoxia and Aerobic Glycolysis. Cancer Res..

[111] Zhang Y., Yu G., Chu H., Wang X., Xiong L., Cai G., Liu R., Gao H., Tao B., Li W. (2018). Macrophage-Associated PGK1 Phosphorylation Promotes Aerobic Glycolysis and Tumorigenesis. Mol. Cell.

[112] Zhang X., Chen L., Dang W.Q., Cao M.F., Xiao J.F., Lv S.Q., Jiang W.J., Yao X.H., Lu H.M., Miao J.Y. (2020). CCL8 secreted by tumor-associated macrophages promotes invasion and stemness of glioblastoma cells via ERK1/2 signaling. Lab. Invest..

[113] Chen F., Chen J., Yang L., Liu J., Zhang X., Zhang Y., Tu Q., Yin D., Lin D., Wong P.P. (2019). Extracellular vesicle-packaged HIF-1alpha-stabilizing lncRNA from tumour-associated macrophages regulates aerobic glycolysis of breast cancer cells. Nat. Cell Biol..

[114] Lin S., Sun L., Lyu X., Ai X., Du D., Su N., Li H., Zhang L., Yu J., Yuan S. (2017). Lactate-activated macrophages induced aerobic glycolysis and epithelial-mesenchymal transition in breast cancer by regulation of CCL5-CCR5 axis: A positive metabolic feedback loop. Oncotarget.

[115] Halbrook C.J., Pontious C., Kovalenko I., Lapienyte L., Dreyer S., Lee H.J., Thurston G., Zhang Y., Lazarus J., Sajjakulnukit P. (2019). Macrophage-Released Pyrimidines Inhibit Gemcitabine Therapy in Pancreatic Cancer. Cell Metab..

[116] Cassetta L., Kitamura T. (2018). Targeting Tumor-Associated Macrophages as a Potential Strategy to Enhance the Response to Immune Checkpoint Inhibitors. Front. Cell Dev. Biol..

[117] Li M., Li M., Yang Y., Liu Y., Xie H., Yu Q., Tian L., Tang X., Ren K., Li J. (2020). Remodeling tumor immune microenvironment via targeted blockade of PI3K-gamma and CSF-1/CSF-1R pathways in tumor associated macrophages for pancreatic cancer therapy. J. Control. Release.

[118] Kaneda M.M., Messer K.S., Ralainirina N., Li H., Leem C.J., Gorjestani S., Woo G., Nguyen A.V., Figueiredo C.C., Foubert P. (2016). PI3Kgamma is a molecular switch that controls immune suppression. Nature.

[119] Kruspig B., Valter K., Skender B., Zhivotovsky B., Gogvadze V. (2016). Targeting succinate:ubiquinone reductase potentiates the efficacy of anticancer therapy. Biochim. Biophys. Acta.

[120] Feichtinger R.G., Lang R. (2019). Targeting L-Lactate Metabolism to Overcome Resistance to Immune Therapy of Melanoma and Other Tumor Entities. J. Oncol..

[121] Su P., Wang Q., Bi E., Ma X., Liu L., Yang M., Qian J., Yi Q. (2020). Enhanced Lipid Accumulation and Metabolism Are Required for the Differentiation and Activation of Tumor-Associated Macrophages. Cancer Res..

[122] Wu L., Zhang X., Zheng L., Zhao H., Yan G., Zhang Q., Zhou Y., Lei J., Zhang J., Wang J. (2020). RIPK3 Orchestrates Fatty Acid Metabolism in Tumor-Associated Macrophages and Hepatocarcinogenesis. Cancer Immunol. Res..

[123] Jin H., He Y., Zhao P., Hu Y., Tao J., Chen J., Huang Y. (2019). Targeting lipid metabolism to overcome EMT-associated drug resistance via integrin beta3/FAK pathway and tumor-associated macrophage repolarization using legumain-activatable delivery. Theranostics.

[124] Oh M.H., Sun I.H., Zhao L., Leone R.D., Sun I.M., Xu W., Collins S.L., Tam A.J., Blosser R.L., Patel C.H. (2020). Targeting glutamine metabolism enhances tumor-specific immunity by modulating suppressive myeloid cells. J. Clin. Invest..

[125] Patel C.H., Leone R.D., Horton M.R., Powell J.D. (2019). Targeting metabolism to regulate immune responses in autoimmunity and cancer. Nat. Rev. Drug Discov..

[126] Leone R.D., Powell J.D. (2020). Metabolism of immune cells in cancer. Nat. Rev. Cancer.

[127] Labadie B.W., Bao R., Luke J.J. (2019). Reimagining IDO Pathway Inhibition in Cancer Immunotherapy via Downstream Focus on the Tryptophan-Kynurenine-Aryl Hydrocarbon Axis. Clin. Cancer Res..

[128] Buck M.D., Sowell R.T., Kaech S.M., Pearce E.L. (2017). Metabolic Instruction of Immunity. Cell.

